# Artificial intelligence chatbots and idiopathic pulmonary fibrosis: are they ready to inform patients?

**DOI:** 10.1590/1806-9282.20251080

**Published:** 2026-04-20

**Authors:** Merve Yumrukuz Şenel, Emine Ayan, Mustafa Çolak, Hikmet Çoban, Fuat Erel, Nurhan Sarıoğlu

**Affiliations:** 1 Balıkesir University, Faculty of Medicine, Department of Chest Diseases – Balıkesir, Türkiye.

**Keywords:** Artificial intelligence, Chatbots, Idiopathic pulmonary fibrosis, Inform, Treatment

## Abstract

**OBJECTIVE:**

The aim of this study was to determine the quality, reliability, and readability of the responses provided by artificial intelligence chatbots about idiopathic pulmonary fibrosis.

**METHODS:**

The clinically relevant questions about idiopathic pulmonary fibrosis diagnosis, treatment, prognosis, and lifestyle management were submitted to four widely used artificial intelligence chatbots, including ChatGPT, Perplexity, Gemini, and Copilot. Responses were assessed by five clinicians based on readability, understandability, quality, and content reliability using standardized tools.

**RESULTS:**

The overall readability of chatbot-generated responses was low, corresponding to high educational requirements. Understandability ranged from moderate to good, whereas actionability remained moderate. Gemini produced the most readable and understandable outputs. Journal of the American Medical Association and Likert scores indicated limited source transparency but good guideline concordance. DISCERN analysis for the treatment question showed significant variation (p=0.003), with Perplexity achieving the highest total score.

**CONCLUSION:**

Although artificial intelligence chatbots offer rapid and accessible information, their readability and source reliability remain limited. These findings highlight the necessity of expert supervision and further model improvement before artificial intelligence chatbots can be safely integrated into patient education.

## INTRODUCTION

Idiopathic pulmonary fibrosis (IPF) is a chronic, fibrosing interstitial pneumonia, typically affecting older individuals, with an unknown etiology^1^. IPF is characterized by progressive worsening of dyspnea and lung function, associated with a very poor prognosis. Although it is a rare disease, its incidence and prevalence appear to be increasing, probably due to increased awareness of the disease^2^.

Patients suspected of having IPF should be referred to specialized centers without delay for accurate diagnosis, appropriate treatment, and management. Two drugs are approved for the treatment of IPF: nintedanib and pirfenidone^3^. It has been shown that both drugs help to slow the progression, improve survival, and reduce lung function decline. Therefore, nintedanib and pirfenidone are introduced as pharmacological treatment options instead of immunosuppressive treatments that have been shown to be ineffective in recent studies^4^. Besides, treatment of common comorbidities such as gastroesophageal reflux disease, long-term oxygen therapy, and lung transplantation should be kept in mind in IPF patients. Additionally, pulmonary rehabilitation involving aerobic conditioning, education and nutrition recommendations, disease awareness, and psychosocial support are important approach to enhance the quality of life of the patients^5^.

Patients obtain information about treatment options from healthcare professionals; however, they often turn to the internet to improve their health and manage their disease. Because IPF is a rare, progressive, and fatal disease with complex diagnostic and therapeutic pathways, patients frequently seek online information due to the disease's poor prognosis, limited treatment options and emotional burden. The internet is a rapidly developing source of every kind of information. Studies have shown that it is frequently used by younger patients, patients suffering from chronic diseases, and those with limited access to healthcare professionals or uninsured patients^6^. Tu et al. stated that internet users tend to think positively and that for many, it has helped them to better understand their disease and treatment, and the information changed their approach to maintaining their health^7^.

Recently, artificial intelligence chatbots (AICs) such as ChatGPT, Gemini, Perplexity, and Copilot have become widely used in people's daily lives due to their ability to solve complex problems through conversations and provide information on various topics, including health problems^8^. Recent developments in AICs have the potential to transform the way patients understand and obtain medical information. Despite the apparent benefits, the application of AICs in healthcare has not been fully investigated^9^. It is crucial to explore its own challenges and limitations, which can complicate its safe, controlled, and effective implementation in healthcare.

While previous studies have evaluated AICs for common diseases such as heart failure, diabetes, and malignancies, no study has evaluated chatbot responses for IPF, a condition where clear and comprehensible communication is vital. Hence, the aim of this study is to compare the responses provided by four AICs and to evaluate the responses in terms of reliability, quality, understandability, and readability by five experts on pulmonary medicine. This aims to clarify how information from AICs may be perceived by the patients and to determine whether AICs are capable of providing accurate, understandable and actionable information to the patients.

## METHODS

This study was conducted in September 2025. The extended set of questions was selected based on the most frequently reported information needs of IPF patients in prior literature and patient advocacy resources. The questions were designed to reflect both clinical and patient-centered aspects of disease understanding, diagnosis, treatment, prognosis, and lifestyle management. The following six questions were used: "what is idiopathic pulmonary fibrosis and what causes it?," "how is idiopathic pulmonary fibrosis diagnosed?," "what treatments are available for idiopathic pulmonary fibrosis?," "what are the side effects of pirfenidone and nintedanib?," "how long can a person live with idiopathic pulmonary fibrosis?," "are there lifestyle changes or supportive treatments that can help manage IPF?." Each question was separately asked to four AICs, including ChatGPT (GPT-4.5, Open AI), Google Gemini (1.0, Google AI), Perplexity (Perplexity AI), and Copilot (GPT-4, Microsoft). All responses were documented on the 24th of September. All queries were entered in English to ensure consistency and comparability. The AICs were accessed using standard, free-to-use versions available to the public. Responses were copied and saved as text files, and no human intervention or manual editing was performed prior to analysis. The responses were evaluated by five senior lecturers in pulmonary medicine (MYS, MÇ, HÇ, FE, and NS) and discrepancies were resolved through consensus. Evaluation included: (1) DISCERN, (2) Journal of the American Medical Association (JAMA), (3) Global Quality Scale (GQS) scores for reliability and quality assessment, (4) Patient Education Materials Assessment Tool (PEMAT) for understandability and actionability, and (5) Automated Readability Index (ARI) for readability of the text. Word counts, sentence counts and punctuation were standardized before applying readability formulas to ensure methodological consistency.

DISCERN is designed to assess the quality of health-related information, and consists of 16 questions scored from 1 to 5; higher scores represent higher quality. It was developed to judge the quality of written information about treatment choices, and it has been proven to be a reliable and valid instrument for this purpose^10^. DISCERN analysis was exclusively applied to the treatment-related query "what treatments are available for idiopathic pulmonary fibrosis?" JAMA was developed by Silberg et al. to evaluate the transparency and publication information of the sources^11^. The scale consists of four questions and is scored from 1 to 4, where one indicates that the data is insufficient, whereas four indicates that the data is completely sufficient. The other score for reliability and quality assessment of internet-based resources is GQS, which is developed by Bernard et al.^12^. The score consists of five questions range from 1 to 5; one indicates poor quality and useless, and five represents the opposite, high quality and highly beneficial information for patients. Guideline concordance and content accuracy were rated using a five-point Likert scale (1=strongly disagree, 2=disagree, 3=neutral, 4=agree, 5=strongly agree). Scores of 4–5 were considered indicative of good or high concordance. PEMAT is another tool to assess the comprehensibility and accessibility of the materials^13^. It consists of 17 questions coded with 0 or 1. A higher score indicates that the material is easily accessible and accurately understandable.

ARI is a readability formula for the evaluation of the understandability of texts. It estimates the educational level required for a reader to understand the text on the first reading. ARI assesses readability based on the average number of characters per word and the average number of words per sentence in a given text sample. By applying a specific formula, the ARI determines the US school grade level required to read a text^14^.

All statistical analyses were performed using the Statistical Package for the Social Sciences (SPSS) v. 25 for Windows (SPSS Inc., Chicago, IL, USA). Descriptive statistics were presented as medians with interquartile ranges (IQRs) for non-normally distributed variables. Comparisons among chatbot responses were performed using the Kruskal-Wallis test. A p-value of <0.05 was considered statistically significant.

## RESULTS

A total of six questions regarding IPF were submitted to the four AICs, and the corresponding responses were summarized in Supplementary Table 1. In terms of readability, the ARI scores significantly differed among chatbots (p=0.011), with Perplexity showing the highest median value (14.9 [13.1–17.3]) and Copilot showed the lowest scores, but still quite difficult to read (12.5 [12–14.4]). For the PEMAT-P understandability score, Gemini achieved the highest score (76.9 [75–82.2]), while Perplexity scored lowest (66.7 [58.3–75]), reflecting relatively more complex or less patient-friendly phrasing. Similarly, PEMAT-P actionability scores differed (p=0.022); Gemini performed better (60 [60–80]) than the others, indicating that its responses were more practical and easier to act on. The results summarized in Table 1.

**Table 1:** Results of readability, understandability and quality assessments of the chatbots.

	ChatGPT	Perplexity	Gemini	Copilot	p-value
ARI	12.7 (11.8–15)	14.9 (13.1–17.3)	14 (11.9–14.6)	12.5 (12–14.4)	**0.011**
PEMAT-P understandability	75 (66.7–76.9)	66.7 (58.3–75)	76.9 (75–82.2)	75 (66.7–75)	**0.001**
PEMAT-P actionability	60 (60–60)	60 (40–66.7)	60 (60–80)	60 (40–60)	**0.022**
GQS	3 (3–4)	3 (2–4)	4 (3–4)	2 (2–3)	**<0.001**
JAMA	0 (0–0)	1 (1–1)	1 (1–1)	0 (0–0)	**<0.001**
Likert scale	5 (4–5)	5 (5–5)	5 (4–5)	4 (4–4)	**<0.001**
DISCERN total	40 (38–43)	55 (53.5–64.5)	46 (44.5–49.5)	51 (44–53)	**0.003**

Data presented as median (interquartile range). Comparisons among chatbot responses were performed using the Kruskal-Wallis test. ARI: Automated Readability Index; PEMAT-P: Patient Education Materials Assessment Tool; GQS: Global Quality Scale; JAMA: Journal of the American Medical Association. Bold values indicate statistically significant p-values (p<0.05).

Regarding content quality, GQS demonstrated marked variation among chatbots (p<0.001). Gemini had the highest GQS (4 [3–4]), while Copilot scored lowest (2 [2–3]). JAMA scores also differed significantly (p<0.001), with Gemini and Perplexity showing higher transparency (1 [1–1]) compared to ChatGPT and Copilot (0 [0–0]), indicating an absence of cited medical sources. Lastly, guideline concordance measured by the five-point Likert scale revealed overall high ratings, but Copilot scored significantly lower (4 [4–4]) compared to others (p<0.001).

Among six evaluated questions, DISCERN analysis was exclusively applied to the treatment-related query "what treatments are available for idiopathic pulmonary fibrosis?," as the DISCERN tool specifically assesses the quality of written information on treatment options. The median DISCERN total scores were 40 (38–43) for ChatGPT, 55 (53.5–64.5) for Perplexity, 46 (44.5–49.5) for Gemini, and 51 (44–53) for Copilot, with statistically significant differences across chatbots (p=0.003). DISCERN scores were summarized in Table 2. These results indicate that chatbot-generated treatment information demonstrated fair to good quality, with Perplexity scoring the highest DISCERN score and ChatGPT performing the lowest among the evaluated AICs.

**Table 2:** DISCERN questions and results for first query.

DISCERN questions 1–8: Assessment of content and reliability		ChatGPT	Perplexity	Gemini	Copilot
1	Are the aims clear?	4 (3–5)	4 (4–5)	5 (4.5–5)	4 (3.5–5)
2	Does it achieve its aims?	4 (3.5–4.5)	5 (4–5)	4 (4–5)	3 (3–3.5)
3	Is it relevant?	5 (4.5–5)	5 (5–5)	5 (4.5–5)	5 (3–5)
4	Is it clear what sources of information were used to compile the publication (other than the author or producer)?	1 (1–1.5)	5 (5–5)	4 (3.5–5)	4 (2.5–4.5)
5	Is it clear when the information used or reported in the publication was produced?	1 (1–1)	1 (1–1)	1 (1–1)	1 (1–1)
6	Is it balanced and unbiased?	4 (3.5–4)	5 (4–5)	4 (4–5)	4 (3.5–5)
7	Does it provide details of additional sources of support and information?	1 (1–1.5)	5 (3–5)	3 (2.5–4.5)	2 (1.5–4)
8	Does it refer to areas of uncertainty?	2 (1–2.5)	2 (2–3.5)	2 (1.5–2)	4 (1.5–4.5)
**Discern questions 9–15: Assessment of details of treatment information**					
9	Does it describe how each treatment works?	2 (1–3.5)	5 (3.5–5)	1 (1–1.5)	4 (3–4)
10	Does it describe the benefits of each treatment?	3 (2.5–3)	4 (2–4)	2 (2–3)	4 (3–4)
11	Does it describe the risks of each treatment?	1 (1–1.5)	4 (3.5–4)	1 (1–1.5)	1 (1–1)
12	Does it describe what would happen if no treatment is used?	1 (1–1.5)	2 (1.5–3)	1 (1–2.5)	2 (1.5–3)
13	Does it describe how the treatment choices affect the overall quality of life?	1 (1–2)	3 (3–4)	2 (1.5–3.5)	3 (2–4)
14	Is it clear that there may be more than one possible treatment choice?	5 (4–5)	4 (3.5–4)	4 (4–4.5)	5 (4.5–5)
15	Does it provide support for shared decision-making?	3 (3–3)	2 (2–3.5)	3 (1.5–3)	2 (2–3)
**DISCERN questions 16: Overall quality assessment**					
16	Based on the answers to all of the above questions, rate the overall quality of the publication as a source of information about treatment choices.	2 (1.5–3)	3 (3–4)	2 (2–3.5)	2 (1.5–3)
	Total score	40 (38–43)	55 (53.5–64.5)	46 (44.5–49.5)	51 (44–53)

Data presented as median (interquartile range).

## DISCUSSION

In recent years, internet-based platforms have become increasingly popular sources of health-related information. Although these platforms can provide valuable content, they also carry the risk of disseminating inaccurate, irrelevant, or misleading information. This concern is particularly significant in the healthcare domain, where public exposure to misinformation can have serious consequences. In this context, our study aimed to evaluate the responses of widely used AICs—whose popularity has grown with recent advancements in large language models—regarding the IPF.

As far as we know, this is the first study to compare AICs, including ChatGPT, Gemini, Perplexity, and Copilot, especially on the subject of IPF, which is a rare disease. Responses generated by chatbots were generally consistent with current clinical guidelines (Likert 4–5), emphasizing the role of antifibrotic therapies, including pirfenidone and nintedanib, for slowing the progression of the disease and managing the symptoms. Supportive care, pulmonary rehabilitation, and lung transplantation for eligible patients were also mentioned. In a study evaluating the appropriateness of ChatGPT responses related to heart failure, the responses were evaluated as reliable and accurate^15^. The study emphasized that if AIC is validated in the future, it could be beneficial for education and providing easily accessible information about heart failure. Another study evaluating AIC responses to respiratory medicine questions revealed that ChatGPT provided correct responses for 63.5% of the questions and incomplete or partially correct responses for 17.5% of the questions^16^. In this study, it was noted that ChatGPT provided extensive information for the questions; however, the responses may contain inaccuracies and may be insufficient when addressing special clinical situations. Similarly, in another report investigating AIC responses to emergency care questions, the authors emphasized that chatbots may frequently provide incomplete or inaccurate information, posing potential risks, particularly for users who are unaware of medical information^17^. Consistent with these findings, our results also indicate that the absence of references and potential lack of up-to-date information in AIC-generated responses represent important limitations that may compromise the reliability of the information provided to patients.

Our results suggest that the readability of the responses was considerably low, making it difficult for individuals across different educational levels to fully comprehend the information provided. In the study evaluating AICs generated responses to cardiopulmonary resuscitation related questions by ChatGPT, Bard, Gemini, and Perplexity, a readability analysis was conducted and, revealing that the responses can be understood by individuals with at least a sixth grade level^14^. In the study conducted by Musheyev et al., responses generated by AICs to frequently asked queries about urologic malignancies were evaluated^18^. While the information provided was generally accurate, the responses were found to be fairly difficult to read, moderately hard to understand and lack clear instructions for users to act on. In contrast to these more common diseases, IPF presents unique informational challenges due to its rarity, complex treatment regimens, and rapidly progressive nature. These factors may exacerbate the communication gap between AIC-generated information and patients’ understanding.

In our study, based on PEMAT-P scores, the understandability was scored as moderate to good, and actionability was assessed as moderate. The frequently used medical terminology in AICs’ responses appears to contribute to lower PEMAT-P scores. The details of treatment information were evaluated with the second part of the DISCERN scores. Although treatment options were mentioned, the responses received low scores due to the lack of clear explanations regarding the potential impact of treatments on quality of life, possible outcomes if treatments are not administered, and treatment-associated side effects. Results for total DISCERN scores revealed moderate for ChatGPT and Gemini, high quality for Perplexity and Copilot. Similarly, GQS scores revealed good quality for Perplexity, poor quality for ChatGPT and Copilot. AICs possess conversational and interactive capabilities, which give them the potential to fill informational gaps through patients’ follow-up queries. One of the limitations of our study is that the evaluation was based on responses to the questions that we determined. In practice, AICs can engage in dialog, allowing patients to seek clarification or additional information, which may influence the overall quality and understandability of the responses. Besides, the responses given by AICs may vary depending on how the questions formulated; variations in questions can lead to different responses. Another limitation of the study is that the responses generated by AICs were evaluated by clinicians, which may not fully reflect patients’ perspectives. As the responses were evaluated solely from the clinicians’ perspective, the findings may not fully reflect the patients’ experience. Patients might prioritize clarity, empathy, and emotional reassurance over medical precision. Including patient feedback in future studies could therefore provide a more comprehensive understanding of how AIC responses are perceived and utilized in real-world contexts.

Furthermore, since all chatbot responses were collected on a single day to ensure comparability among models, potential temporal variability due to subsequent model updates could not be assessed. Future studies conducted at multiple time points could provide insight into the consistency and evolution of AIC outputs over time. Additionally, although the evaluations were performed independently by five pulmonologists and finalized by consensus, formal inter-rater reliability metrics were not calculated. Incorporating such measures in future research could strengthen the objectivity of findings.

## CONCLUSION

In conclusion, IPF is a life-threatening disease with high mortality, prompting many patients to seek information about their condition on the internet. AICs offer clear advantages in this context with their speed and ability to provide information through interactive dialog. However, our findings highlight important limitations about AICs usage by the patients. Especially in complex clinical scenarios, AICs may produce misleading or incomplete guidance, and their responses often have low readability and limited comprehensibility. Moreover, the failure to cite reliable medical sources further limits their reliability. Although the potential harms associated with misinformation were not quantitatively assessed in this study, these findings highlight the necessity for cautious implementation under the supervision of medical professionals. Future research should aim to validate and refine chatbot performance through the inclusion of empirically tested visual or multimodal components, improved language clarity, and explicit citation of evidence-based sources to enhance the accuracy, transparency, and educational value of artificial intelligence (AI)-generated medical information.

## Data Availability

The datasets generated and/or analyzed during the current study are available from the corresponding author upon reasonable request.
